# Maternal macronutrient and energy intake during pregnancy: a systematic review and meta-analysis

**DOI:** 10.1186/s12889-024-17862-x

**Published:** 2024-02-15

**Authors:** Mohammad Khammarnia, Alireza Ansari-Moghaddam, Fatemeh Govahi kakhki, Cain Craig Truman Clark, Fatemeh Bagher Barahouei

**Affiliations:** 1 Health Promotion Research Center, Zahedan University of Medical Sciences, Zahedan, Iran; 2 Student research Committee, Zahedan University of Medical Sciences, Zahedan, Iran; 3 Exercise and Life Sciences, Coventry University, Coventry, England, UK

**Keywords:** Maternal nutrition, Pregnancy, Maternal health, Child health, Macronutrient, Energy

## Abstract

**Background:**

Nutritional status during pregnancy can have a significant impact on infant and maternal health outcomes. To maintain maternal homeostasis and support fetal growth, adequate macronutrient and energy intake during pregnancy is essential. Therefore, this study sought to systematically review and meta-analyze macronutrient and energy intakes during pregnancy.

**Methods:**

A systematic review and meta-analysis was carried out based on the PRISMA (Preferred Reporting Items for Systematic Reviews and Meta-Analyses) guidelines. The required data were collected from four databases including: Web of Sciences, ProQuest, Scopus, and PubMed, from 1 January 1980 to 30 May 2023, by using a combination of search terms (dietary pattern" OR "diet quality" OR "food habits" OR "nutrition surveys" OR "diet surveys" OR "food-frequency questionnaire" OR "diet record" OR "dietary recall") AND ( "pregnancy" OR "reproduction" OR "maternal health" OR "neonatal outcomes") among interventional and observational studies. Excel and STATA version 11 were used for data analysis.

**Results:**

Among 7081 published articles, 54 studies were included in the review. Most of the 33 (61%) studies were cohort studies and a total of 135,566 pregnant women were included. The overall average of energy, carbohydrate, fat, and protein intake was 2036.10 kcal/day, 262.17 gr/day, 74.17 gr/day, and 78.21 gr/day, respectively. Also, energy intake during pregnancy was higher in American (2228.31 kcal/day, CI95%: 2135.06–2325.63) and Eastern Mediterranean regions (2226.70 kcal/day, CI95%: 2077.23–2386.92) than other regions (*P* < 0.001). Energy intake was higher in the third trimester than others (2115.64 kcal/day, CI95%: 1974.15–2267.27). Furthermore, based on the findings, there was a significant difference between energy intake in different World Health Organization (WHO) regions (*P* < 0.05).

**Conclusions:**

According to the results of meta-analysis, the average total energy was below than average total energy required during pregnancy. More efforts are needed to encourage women to adopt healthy eating habits during pregnancy to support healthy fetal and infant development.

**Supplementary Information:**

The online version contains supplementary material available at 10.1186/s12889-024-17862-x.

## Introduction

Nutrient status, as well as adequate dietary intake, during the human gestational period is essential to ensure optimal fetal growth [1]. Nutrient requirements are enhanced during the pregnancy in all the three trimesters, yet scientific evidence shows pregnant women are at increased risk of micro- and macronutrient deficiency [2].

Macronutrients (protein, fat and carbohydrates) are the main sources of energy for the mother and the fetus, which are necessary for tissue growth and fetal cells development [3]. Pregnancy is associated with increased requirements for nutrition intake and maternal energy in order to meet nutritional demands of the developing fetus.

Inadequate diets resulting in nutrient and energy intake deficiencies can have a considerable impact on neonatal health and pregnancy outcomes**.** Restriction of energy and nutrition disrupt proper development of the fetus and may lead to ailments, including cardiovascular disease, type II diabetes, and hypertension [4]. Chronic energy deficiency commonly occurs among women of reproductive age, as well as pregnant women in developing countries, due to several factors such as poor diet quality, family size, living in rural areas, insufficient meal frequency, and low socioeconomic status [5, 6]. Furthermore, a prior study reported a positive association between energy intake at the end of the third trimester and neonate birth weight. Sharma et al. indicated that higher consumption of carbohydrates may be associated with an increase in birth weight and conversely, increasing fat intake with low birth weight [7]. Additionally, another study showed that reducing dairy, as well as animal proteins, consumption in late pregnancy and enhancing carbohydrate intake in early pregnancy was associated with decreased birth weight [8]. Animal studies have indicated that insufficient dietary protein intake during pregnancy produced offspring with low birth weight [9]. Accordingly, the collective evidence highlights that macronutrients are essential for optimal development of the fetus. Therefore, the aim of the current study was to conduct and systematic review and meta-analysis of maternal macronutrient and energy intake during pregnancy**.**

## Methods

A systematic and meta-analysis study was carried out in 2023. Relevant databases including PROQUEST, SCOPUS, PUBMED and Web of Sciences were searched to identify studies.

### Search strategy

The following search terms were used: ("*dietary pattern”)* OR (*"diet quality"*) OR ( *"food habits"*) OR ( *"nutrition surveys"*) OR ( *"diet surveys"*) OR ( *"food-frequency questionnaire"*) OR ( *"diet record"*) OR ( *"dietary recall"*) AND ( *pregnancy*) OR (*"* *reproduction"*) OR ( *"maternalhealth"*) OR ( *"neonatal outcomes"*). The search terms for each database is shown in Appendix 1.

Inclusion criteria were studies that reported mean or median of energy intake and the percentage or grams of macronutrient (protein, fat, carbohydrate) in healthy pregnant women without having a disease, use food frequency questioner (SO-FFQ), dietary recall, or food dairy(FD) questioners.

Exclusion criteria were: studies that did not report energy, macronutrient (protein, fat, carbohydrate) in healthy pregnant women and countries with special socioeconomic conditions.

Studies were excluded if they were published in a language other than English, examined different specific dietary patterns (such as western, vegetarian, traditional and mixed, prudent and etc.…), did not report the average total macronutrient and energy or diet data included supplements and studies with incomplete information, studies in which women were pregnant under certain conditions (such as IVF). Also, case reports, case series, editorials, letters to the editor, commentaries, and reviews were excluded.

### Data extraction

Study characteristics were extracted into a predetermined table in the Excel software that collected information including author, year of publication, participant number, study design, country, average age, trimesters of pregnancy, dietary assessment tools, average of total macronutrient and energy intake.

**Fig. 1:**
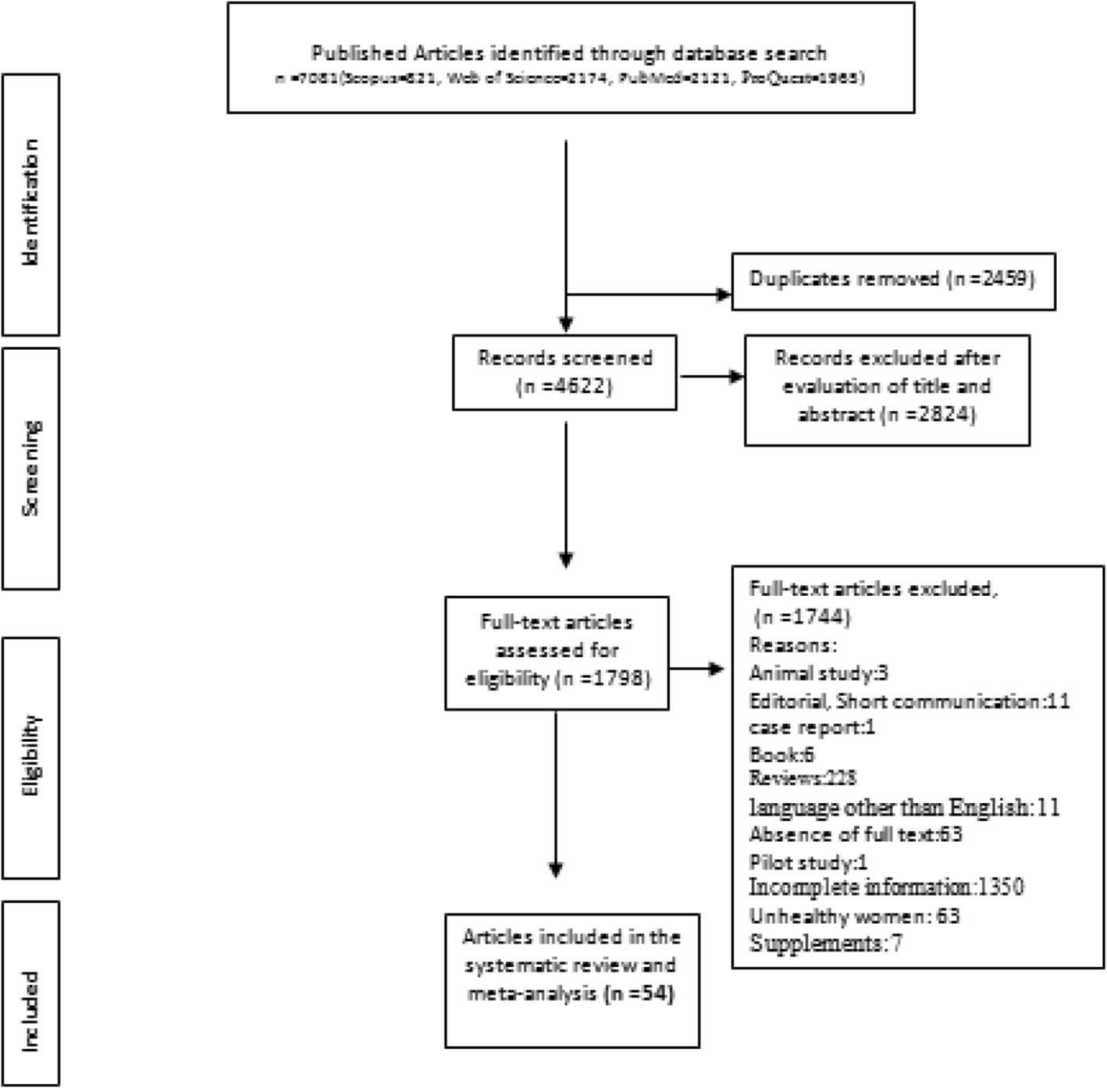
PRISMA flow diagram for the systematic review and Meta_analysis

A total of 54 papers were included in the meta-analysis and all relevant data were extracted. Supplementary data are shown in Fig. 1. Mean energy intakes were extracted from the studies. Also, SD and confidence interval’s (CI) were calculated using the following approach.

### Calculating confidence intervals

The upper limit and lower limit were calculated using the following formula [10, 11].

$$\overline{{\text{X}}}\pm {\text{Z}}\times \frac{\sigma }{\surd {\text{n}}}$$

*X̄ is the sample mean, σ is the standard deviation, and n is the sample size. Assuming a confidence level of 95%: Z = 1.960.

All energy units, except kilocalories, were converted to kilocalories. When macronutrient percentages were given, we converted them to grams if they could be converted manually, otherwise they were excluded.

gr/day carbohydrate or protein = Energy * % carbohydrate or protein / 4 and for fat it is 9.

We estimated the sample mean from the sample size, median, mid-range, and/or mid-quartile range [12]:

In the studies where the sample quartile was given, the mean and standard deviation were calculated using the following formula. Also, studies that reported median information were converted to the mean [13, 14].

$$\overline{{\text{X}}}\approx \frac{{\text{q}}1+m+{q}_{3}}{3}$$

*Scenario S2 reports the first and third quartiles instead of the minimum and the maximum, together with the median and the sample size.

standard deviation:

The standard error of the mean (SE) was converted to the standard deviation(SD) using the formula [15].

SD = SE *√n.

*n* = sample size.

Decimals of the mean and standard deviation were rounded.

However, some macronutrients were not reported within the included studies.

### Statistical analysis

Mean of macronutrient and energy intake pooled odds were calculated with a random-effect restricted maximum likelihood (REML) model and 95% confidence intervals. We inferred heterogeneity between studies using the I^2^ statistic. By using subgroup analysis, and taking into account region, questionnaire, trimester of pregnancy, and study design, we assessed the heterogeneity of studies. Publication bias was assessed using Egger's test. All data analysis was carried out using STATA software.

## Results

All articles were imported into Endnote software version 16, where duplicates were removed. Subsequently, the title and abstract of articles were studied and they were included if were relevant to the topic. Following this, the authors read the full text, and eligible studies with selection criteria were obtained. Briefly, the flow diagram for the studies selection is presented in Fig. 1.

As shown in the PRISMA flow diagram (Fig. 1), 7081 published papers were identified from (Web of Sciences, ProQuest, Scopus, and PubMed) in 1 January 1980 to 30 may 2023, of which, 2459 cases were duplicates and removed. After reading the titles and abstracts, 2824 articles were excluded due to being unrelated to the topic. Also, 1744 articles were excluded after reviewing the full texts of remaining articles. Therefore, a total of 54 studies were included in this review. Two of the studies were randomized controlled trial (RCTs), 33 were cohort, 15 studies were cross-sectional, and 4 of them were case–control studies. The basic characteristics of the included studies are presented in Table 1. The age of the participants ranged from 23 ± 3 (years) [16], to 37 ± 4 years [17]. Out of the total studies included, most of the studies were conducted in America [18–40]. Additionally, four studies were from the Eastern Mediterranean region [41–44], followed by 13 from the western pacific region [16, 45–56], 11 from European region [17, 37, 57–66], and two were conducted in South East Asia [67, 68]. In total, the sample size was 135,566 participants, ranging from 28 in Lebrun [24] to 92,448 in Miura [69]. Only 24 studies used the dietary recall method, and most of the dietary assessments were realized using the food frequency questionnaire, whilst 11 used foods dairy questionnaire.

**Table 1:** Description of the studies included in the meta-analysis

**Author**	**Study design**	**Country**	**Sample size**	**age (years)**	**trimesters of pregnancy (Month)**	**Dietary Assessment Tool**	**Energy(Mean,SD)**	**Protein (gr/d)** **(Mean,SD)**	**Carbohydrates** **(gr/d)(Mean,SD)**	**Fat (gr/d)** **(Mean,SD)**	Quality Study
Li,2022 [45]	Cross-sectional	China	7347	N/A	1–3	FFQ	2323 ± 781	70 ± 31	345 ± 124	88 ± 37	High
Gete,2021 [49]	Cohort	Australian	621	33 ± 1	1–3	FFQ	1707 ± 516	N/A	N/A	N/A	High
De La Rosa,2020 [18]	Cohort	Navajo	242	28 ± 6	3	FFQ	2213 ± 1012	95 ± 48	285.5 ± 150	81 ± 41	Medium
Perreault,2016 [70]	Cohort	Canada	42	31 ± 4	3	FFQ, FD	N/A	FFQ:70 ± 25 FD:83 ± 22	N/A	N/A	Medium
Ancira-Moreno,2020 [19]	Cohort	Mexico City	660	25 ± 6	2,3	24 h-R	2333 ± 638	98 ± 28	265 ± 83	85 ± 35	Medium
Miura,2020 [53]	Cohort	Japan	92,448	31 ± 5	1	FFQ	1830 ± 823	N/A	N/A	N/A	Medium
Hu,2020 [20]	Cohort	Tennessee	1257	26 ± 5	2	FFQ	2726 ± 1666	N/A	N/A	N/A	High
Baddour,2013 [21]	Case–control	Canada	45	29 ± 5	3	FFQ	2425 ± 663	107 ± 33	322 ± 94	85 ± 25	High
Mahmassani,2021 [22]	Cohort	Massachusetts	1580	32 ± 5	1, 2	FFQ	2061 ± 693	N/A	N/A	N/A	Medium
Yang,2019 [46]	Case–control	China	948	N/A	1–3	FFQ	2045 ± 954	N/A	N/A	N/A	Medium
Duarte,2020 [36]	Cross-sectional	Brazil	155	29 ± 1	2 to 3	FFQ and 24 h-R	FFQ:3307 ± 1379 24 h-R:1980 ± 600	106 ± 34 87 ± 27	289 ± 92	65 ± 28	Medium
Shatenstein,2011 [23]	RCT	Mexico	107	31 ± 5	2	FFQ, FD	FFQ:1963 ± 610 FD: 2320 ± 607	91 ± 29 98 ± 28	237 ± 83	77 ± 28	Medium
Cole,2008 [71]	Cohort	UK	198	27 ± 5	1,3	FFQ	N/A	85 ± 5	N/A	N/A	Low
Ogawa,2017 [50]	Cohort	Japanese	188	35 ± 4	1	FFQ, FD	FFQ:1744 ± 560 FD:1643 ± 403	59 ± 22 60 ± 17	228 ± 57	53 ± 19	Medium
Savard,2018 [25]	Cross-sectional	Quebec City	60	32.5 ± 3.5	2	24 h-R,FD	24 h-R:2357 ± 489 FD:2239 ± 506	100 ± 20 98 ± 23	279 ± 76	85 ± 23	Medium
Voortman,2020 [33]	Cohort	Dutch	83	32 ± 4	2	FFQ, 24 h-R	FFQ:2149 ± 1246 24 h-R:2162 ± 982	75 ± 12.5 78 ± 10	249 ± 43	81 ± 14	Medium
Landman,1989 [26]	Cohort	Jamaica	108	N/A	1–3	24 h-R	2110 ± 739	73 ± 35	N/A	N/A	Medium
Apostolopoulou,2021 [64]	Cross-sectional	Greece	70	34 ± 5	2	FFQ, 24 h-R	24hR:2124 ± 2179 FFQ:2294 ± 1975	66 ± 17 65 ± 17	186 ± 61	88 ± 35	Medium
Forsythe, 1994 [35]	Cross-sectional	KENTUCKY/OHIO	80	30 ± 5	3	FFQ, 24 h-R	24hR: 1919 ± 569 FFQ: 2573 ± 440	91 ± 51 120 ± 48	260 ± 125	86 ± 59	Medium
BROWN,1996 [29]	Cohort	Minnesota	56	32 ± 3	**2**	FD, FFQ	FD:2258 ± 344 FFQ:2031 ± 613	88 ± 15 89 ± 28	270 ± 85	**70** ± 24	High
Vilela,2017 [37]	Cohort	England	12,195	N/A	3	FFQ	1672 ± 478	69 ± 20	212 ± 63	72 ± 23	High
Brantsæter, 2008 [65]	Cohort	Norway	119	31 ± 4	2	FFQ, FD	FFQ:1150 ± 716 FD:1150 ± 478	87 ± 14 81 ± 10	271 ± 35	76 ± 15	Medium
McGowa2013 [58]	RCT	Ireland	130	32 ± 4	2.3	FFQ, FD	FFQ: 2153 ± 717 FD: 1914 ± 478	96 ± 35 78 ± 15	236 ± 60	78 ± 23	High
McGowan, 2012 [72]	Cohort	Ireland	285	N/A	1–3	FD	1914 ± 478	81 ± 9.5	239 ± 24	77 ± 8.5	High
Hinojosa-Nogueira,2021 [59]	Cohort	Spain	690	31 ± 5	1,3	FFQ	2052 ± 671	90 ± 29	230 ± 84	94 ± 37	Medium
Schwedhelm,2022 [27]	Cohort	United States	365	31 ± 5	1–3	24 h-R	2047 ± 657	N/A	N/A	N/A	Medium
Lebrun, 2019 [24]	Cohort	Canada	28	33 ± 4	3	24 h-R	2321 ± 429	100 ± 19	283 ± 64	92 ± 23	Low
Angkasa, 2019 [67]	Cross-sectional	Indonesian urban	100	28	3	FFQ, 24 h-R	FFQ:2025 ± 635 24 h-R:2186 ± 527	N/A	N/A	64 ± 31	High
Pinto, 2010 [60]	Cohort	Portugal	101	30 ± 5	1–3	FD	FD:2171 ± 388	100 ± 16	262 ± 52	82 ± 18	High
Darke, 1980 [61]	Cross-sectional	Great Britain	435	N/A	2	FD	2152 ± 503	70 ± 17	260 ± 69	98 ± 26	Low
Gao, 2013 [47]	Cross-sectional	China	192	N/A	3	24 h-R	2338 ± 844	69 ± 27	281 ± 104	106 ± 58	Low
Pick,2005 [32]	Case–control	Canada	52	30 ± 1	3	FD	2309 ± 371	86 ± 14	323 ± 35	74 ± 42	Medium
Zhang, 2015 [55]	Cohort	Chinese	123	28 ± 4	1,2	FFQ. 24-hR	FFQ:1748 ± 400 24HR:1717 ± 415	57 ± 18 58 ± 18	246 ± 57	52 ± 10.5	Medium
Lepsch, 2014 [38]	Cohort	Brazil	248	27 ± 1	1	FFQ	2219 ± 477	93 ± 1	325 ± 35	69 ± 11	Medium
Emond, 2018 [28]	Cohort	New Hampshire	862	N/A	3	FFQ	2086 ± 672	N/A	N/A	N/A	Medium
Yang,2016 [54]	Cross-sectional	China	7462	N/A	1-3 m	FFQ	2070 ± 100	62 ± 4	301 ± 13	79 ± 4	Medium
Joshi, 2018 [68]	Cohort	India	75	27 ± 0.49	3	24-hR	1729 ± 53	53 ± 2	N/A	66 ± 22.5	Low
Cheng,2008 [56]	Cohort	China	125	25	1–3	24 h-R, FFQ	FFQ:1903 ± 435 24HRs:1673 ± 330	48 ± 12 44 ± 9	316 ± 64	32 ± 10	Medium
Chen, 2017 [51]	Cohort	Singapore	1048	30.5 ± 5	3	FFQ	1846 ± 562	74 ± 18	240 ± 41.5	67 ± 16	Medium
LI,2013 [16]	Cohort	China	168	23 ± 3	3	24 h-R	1654 ± 394	77 ± 25	272 ± 63	34 ± 16	Medium
Dubois, 2018 [34]	Cohort	Canada	861	N/A	1–3	FD	2232 ± 390	92 ± 18	288 ± 57	83 ± 21	Medium
Athanasiadou, 2016 [17]	Cross-sectional	Greece	179	37 ± 4	2–3	FFQ, 24 h-R	FFQ:1838 ± 271 R:1806 ± 310	69 ± 11 70 ± 16	170 ± 41	89 ± 17	Medium
Tayyem,2019 [44]	Cohort	Jordan	131	30 ± 5	1–3	24 h-R,FFQ	R:2235.5 ± 382 FFQ:2492 ± 408	85 ± 17 82 ± 16	328 ± 49	97 ± 24	Medium
Mohammadshahi,2013 [41]	Cross-sectional	Iran	94	26 ± 6	3	FFQ	2268 ± 423	89 ± 22	294 ± 90	83 ± 18	Medium
Reyes-López, 2021 [39]	Cohort	Mexican	226	29 ± 8	2	24 h-R	1815 ± 123	80 ± 23	234 ± 18	60 ± 5	High
Li,2020 [48]	Cohort	China	404	31 ± 5	N/A	24 h-R	1997 ± 727	63 ± 29	260 ± 45	80 ± 18	Low
Gonzalez-Nahm,2022 [30]	Cross-sectional	Carolina	468	**27 ± 5.5**	**2, 3**	FFQ	2174 ± 945	N/A	N/A	N/A	Medium
Mouratidou,2006 [66]	Cross-sectional	UK	123	29 ± 6	2	24 h-R, FFQ	FFQ:1923 ± 516 24-Hour recall: 1546 ± 370	70 ± 20.5 55 ± 15	196 ± 56	65 ± 20	Medium
Papazian, 2016 [42]	Cross-sectional	Lebanese	128	29 ± 5	1–3	24 h-R, FFQ	24 h recall:2003 ± 431 FFQ:2381 ± 565	75 ± 18 87 ± 23	255 ± 59	85 ± 67	Medium
Alamolhoda,2019 [43]	Case–control	Iran	407	**25 ± 3**	1–3	24 h-R	2029 ± 101	N/A	N/A	N/A	Medium
Shin,2016 [31]	Cross-sectional	USA	343	N/A	**1**	24 h-R	2246 ± 83	81 ± 37	298 ± 104	N/A	Medium
Vioque,2013 [63]	Cohort	Spain	740	N/A	**1,3**	FFQ	2258 ± 610	100.5 ± 26	257 ± 83.5	96 ± 30	Medium
Loy,2011 [52]	Cohort	Malaysia	177	30	2,3	24 h-R	1822 ± 324	70 ± 12	249 ± 49	57 ± 13	Medium
Vilela,2014 [40]	Cohort	Brazil	248	27	1,2	FFQ	2250	N/A	N/A	N/A	Medium

### Study quality assessment

The CASP^*^ checklist was used to evaluate the quality of the studies. In total, 54 studies entered the meta-analysis phase. These studies are shown in Table 1.

* The CASP (Critical Appraisal Skills Program) checklists are a set of checklists that contain prompt questions to help evaluate research studies.

### Total protein intake

**Fig. 2:**
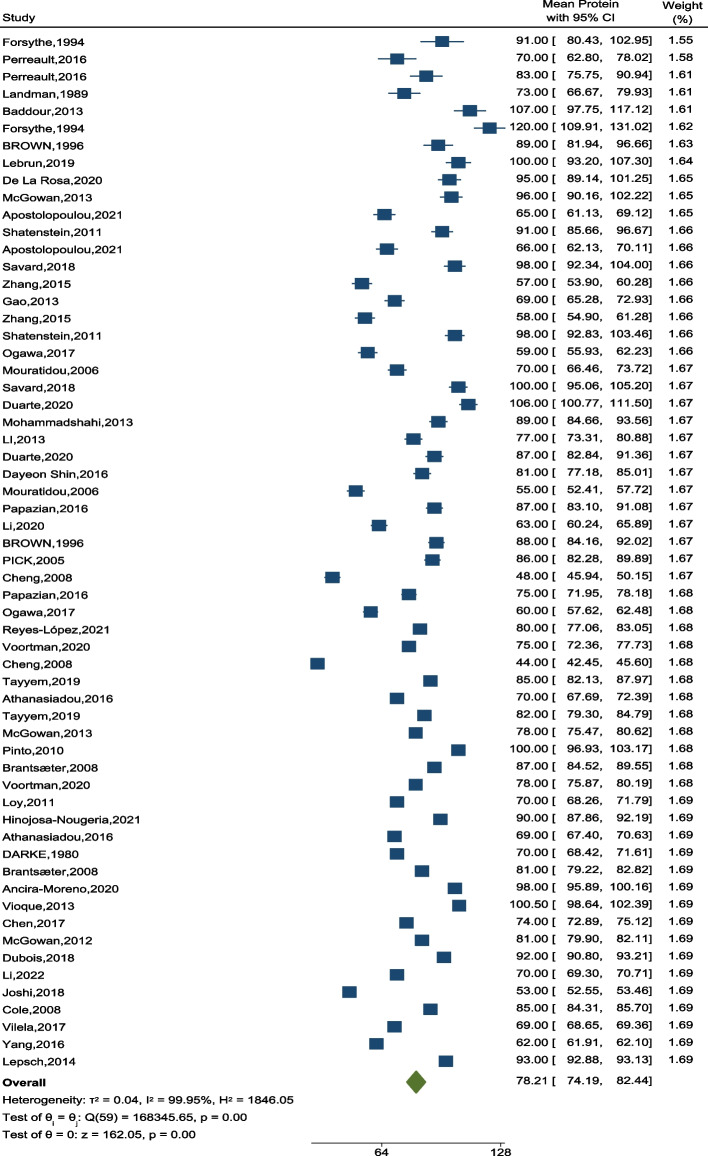
The forest plot of the overall mean of protein

Analysis showed that total protein intake amongst pregnant women in different countries, albeit with different assessment instruments of food intake, was 78.21 gr/day (95% CI: 74.19–82.44) (Fig. 2).

The highest of daily energy intake from protein was found in Forsythe study (19–25% According to the difference of energy in recall and FFQ questionnaires). According to the results, there was a significant difference between the amount of protein consumed during pregnancy in different studies (*p* < 0.001).

### Total carbohydrate intake

**Fig. 3:**
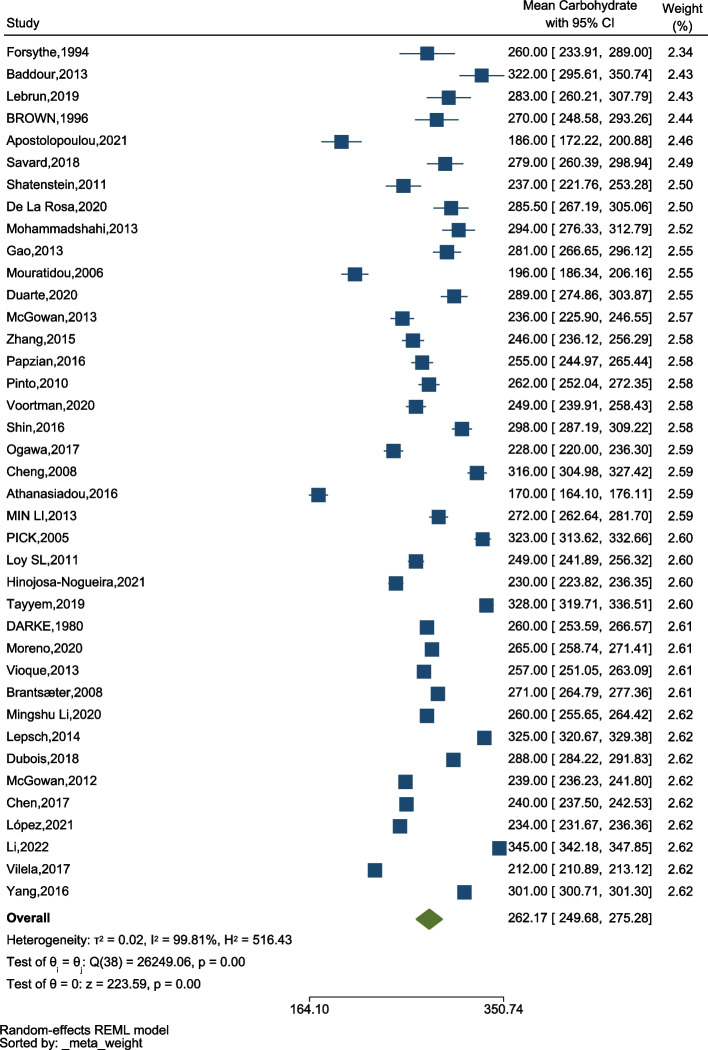
The forest plot of the overall mean of carbohydrates

All countries showed a high intake of carbohydrate, ranging from 170gr/day in Greece (95% CI: 164.10–176.11) to 345gr/day (95% CI: 342.18–347.85) in China (Fig. 3). Also, there was a significant difference between the amount of carbohydrate intake during pregnancy in different studies (*p* < 0.001).

### Total fat intake

**Fig. 4:**
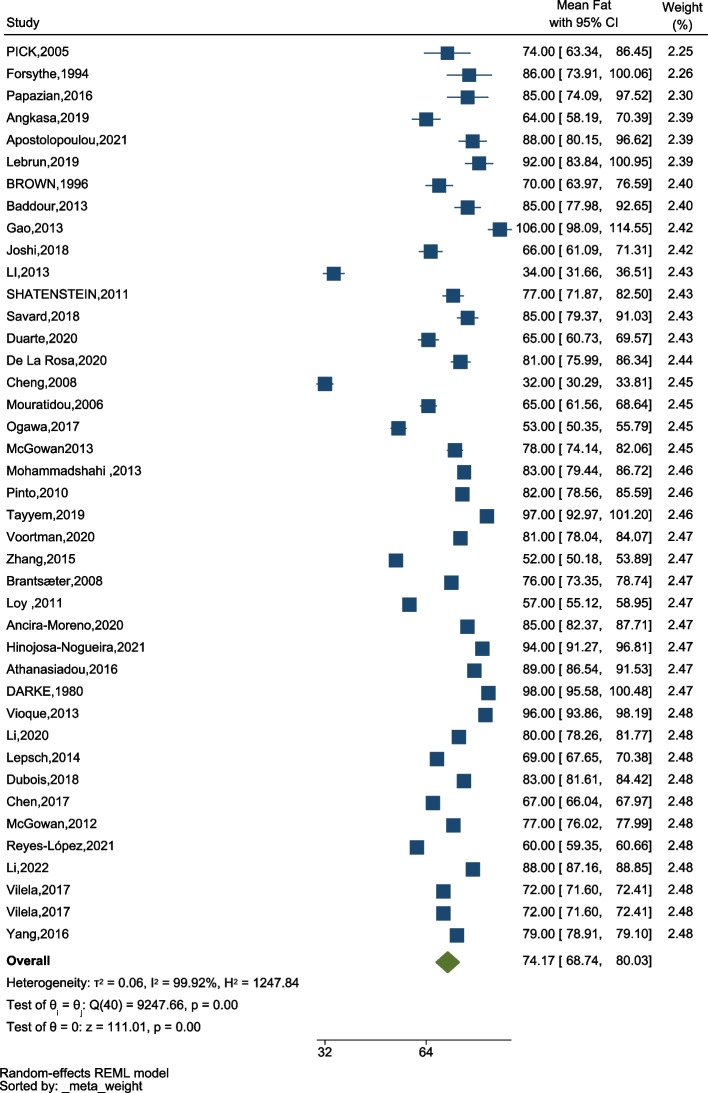
The forest plot of the overall mean of fat

Across the WHO regions, total fat intake amongst pregnant women was 74.17 gr/day (95% CI: 68.74–80.03), the lowest amount of fat intake was in the Cheng study conducted in China 32 gr/day (95% CI: 30.29–33.81), and the highest intake was in the Gao study conducted in China in 2013 (106 gr/day, 95% CI: 98.09–114.55) (Fig. 4). According to our results, there was a significant difference between the amount of fat consumed during pregnancy in different studies.

### Energy intake

**Fig. 5:**
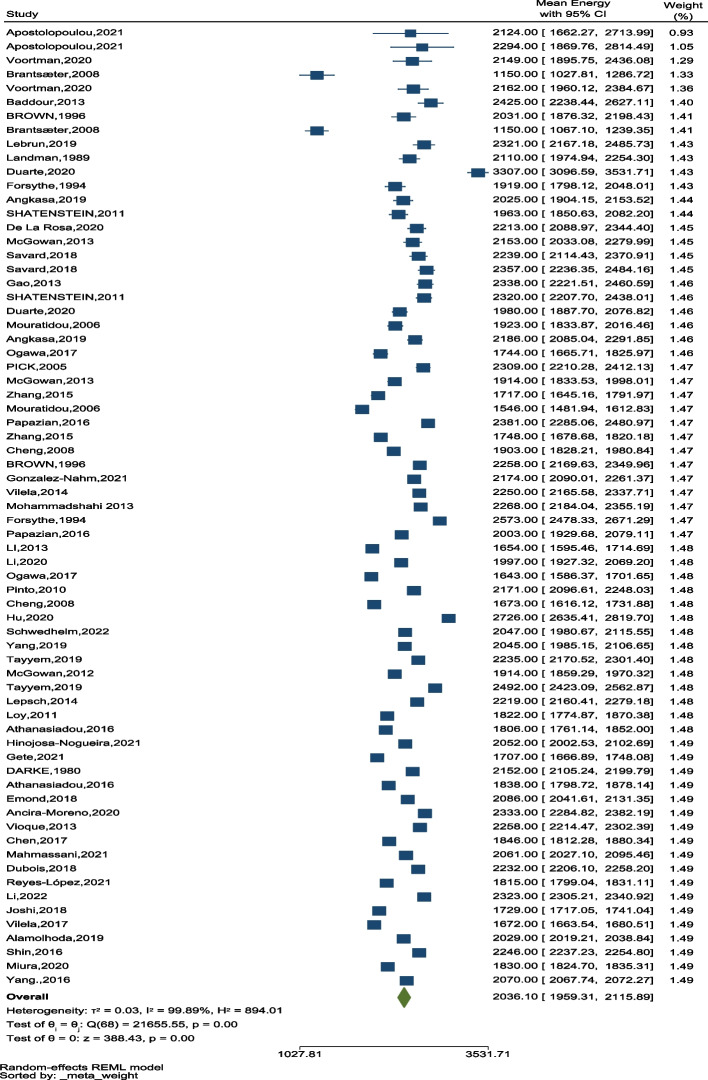
The forest plot of the overall mean of energy

Among 54 studies, data on energy intake was extracted from 52 studies. The overall average energy intake was 2036.10 kcal/day (95% CI: 1959.31–2115.89), ranging from 1150 kcal/day (95% CI: 1027.81–1286.72) in Brantsæter.et al. to 3307 kcal/day (95% CI: 3096.59–3531.71) in the Duarte study (Fig. 5). We found a significant difference between the amount of energy intake during pregnancy.

### Energy intake

**Fig. 6:**
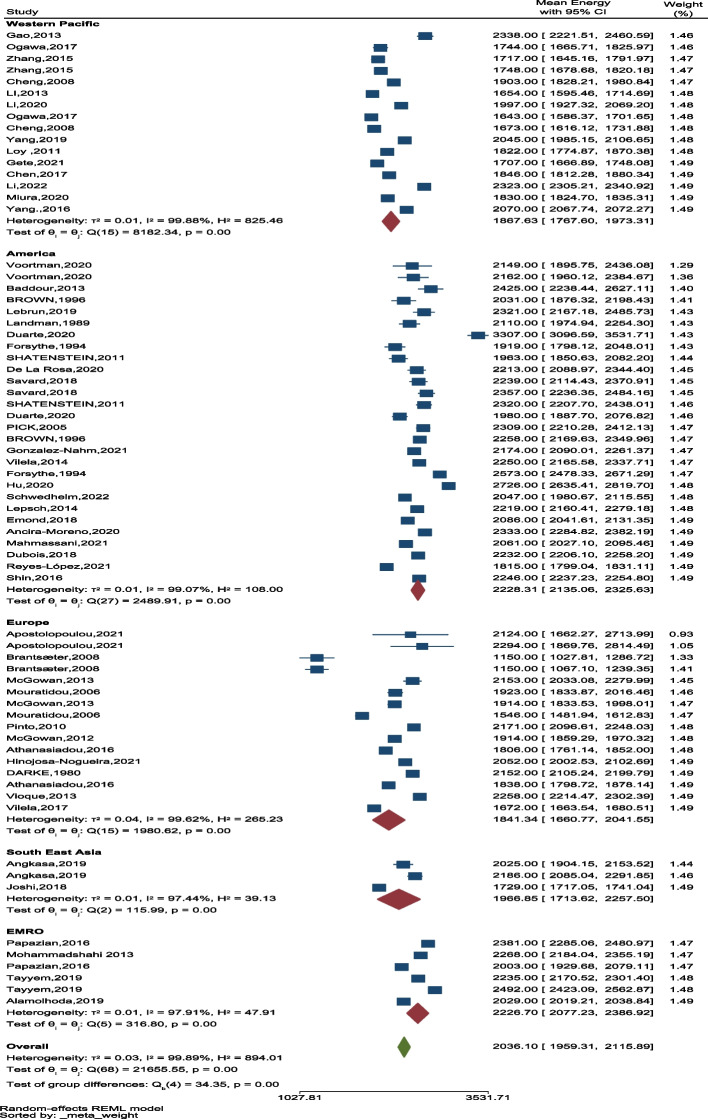
The forest plot of average energy in 5 geographical areas

By contrasting levels of intake of energy in countries, we found that participants from Mexico had a higher daily energy intake than those from Norway. Based on the division of geographical regions into five regions, the highest average energy was seen in the American and Eastern Mediterranean regions and the lowest in the Western Pacific regions (Fig. 6).

**Fig. 7:**
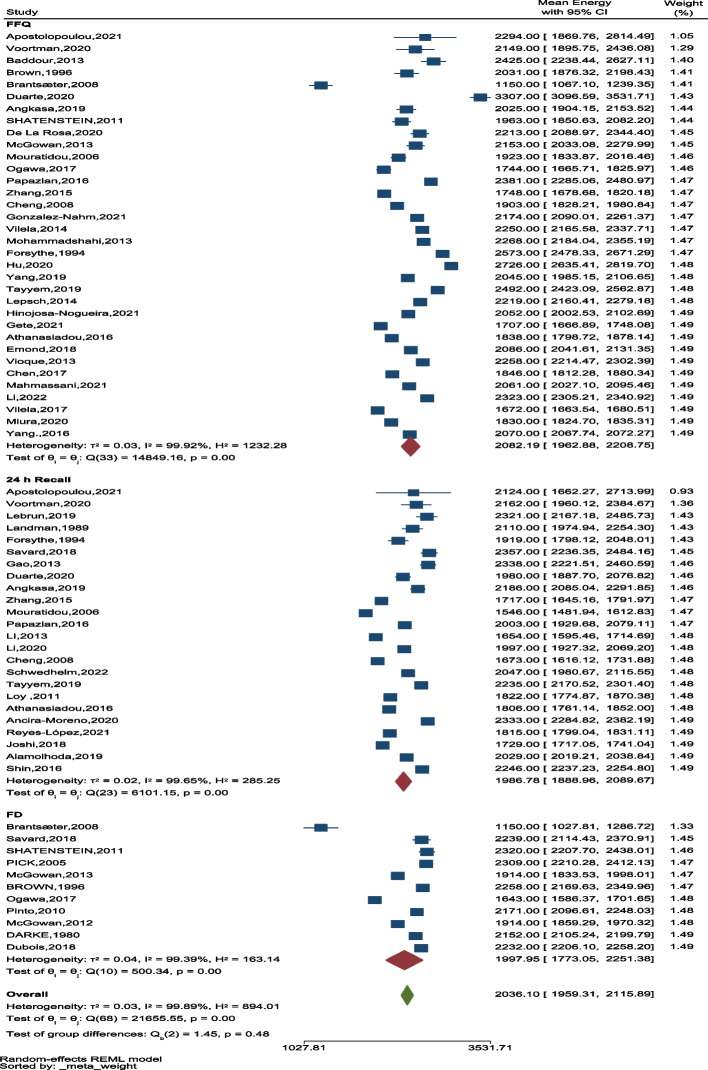
The forest plot of the overall mean of energy based on three questioners

According to Table 2, the average energy using the questionnaires were significantly different, although higher values were observed in studies using the FFQ questionnaire (Fig. 7).

**Table 2:** Energy classification based on the type of questionnaire, geographical region, type of study, duration of pregnancy

	**Classification**	**Total mean**^**a**^	**CI 95%**	***P*-value**
**Macronutrient**	Carbohydrate	262.17	249.68–275.28	< 0.001
	Protein	78.21	74.19–82.44	< 0.001
	Fat	74.17	68.74–80.03	< 0.001
**Questioner**	24-h recall	1986.78	1888.96–2089.67	< 0.05
	FD	1997.95	1773.05–2251.35	
	FFQ	2082.19	1962.88–2208.75	
**WHO Region**	Eastern Mediterranean region	2226.70	2077.23–2386.92	
	Western pacific region	1867.63	1767.60–1973.31	< 0.001
	Region of the America	2228.31	2135.06–2325.63	
	European region	1841.34	1660.77–2041.55	
	South-east Asia region	1966.85	1713.62–2257.53	
**Study design**	Case–control	2243.58	2030–2479.63	< 0.05
	Cohort	1950.49	1845.51–2061.43	
	Cross-sectional	2151.82	2025.03–2286.54	
	RCT	2069.27	1935.19–2212.64	
**Trimester of pregnancy**	First trimester	1957.81	1706.01–2182.79	0.50
	Second trimester	1978.98	1801.02–2174.53	
	Third trimester	2115.64	1974.15–2267.27	
	Total trimester	2024.60	1947.54–2104.69	

^**a**^Weighted mean with 95% confidence interval (CI), *P*-value < 0.05

**Fig. 8:**
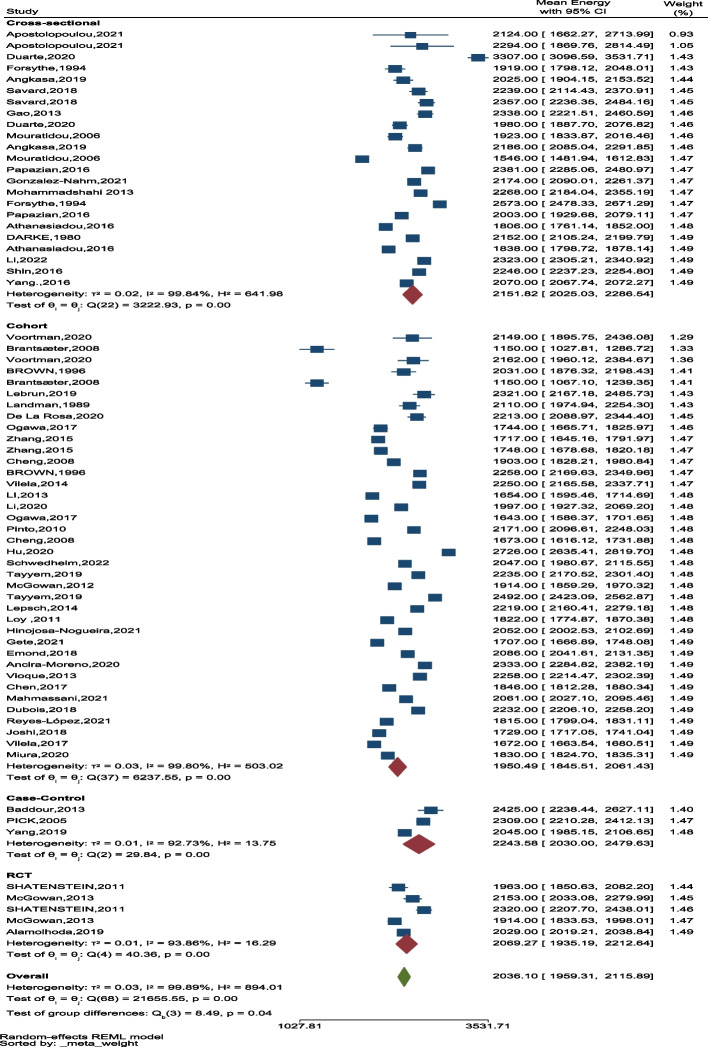
The forest plot of the overall mean of energy based on study design

**Fig. 9:**
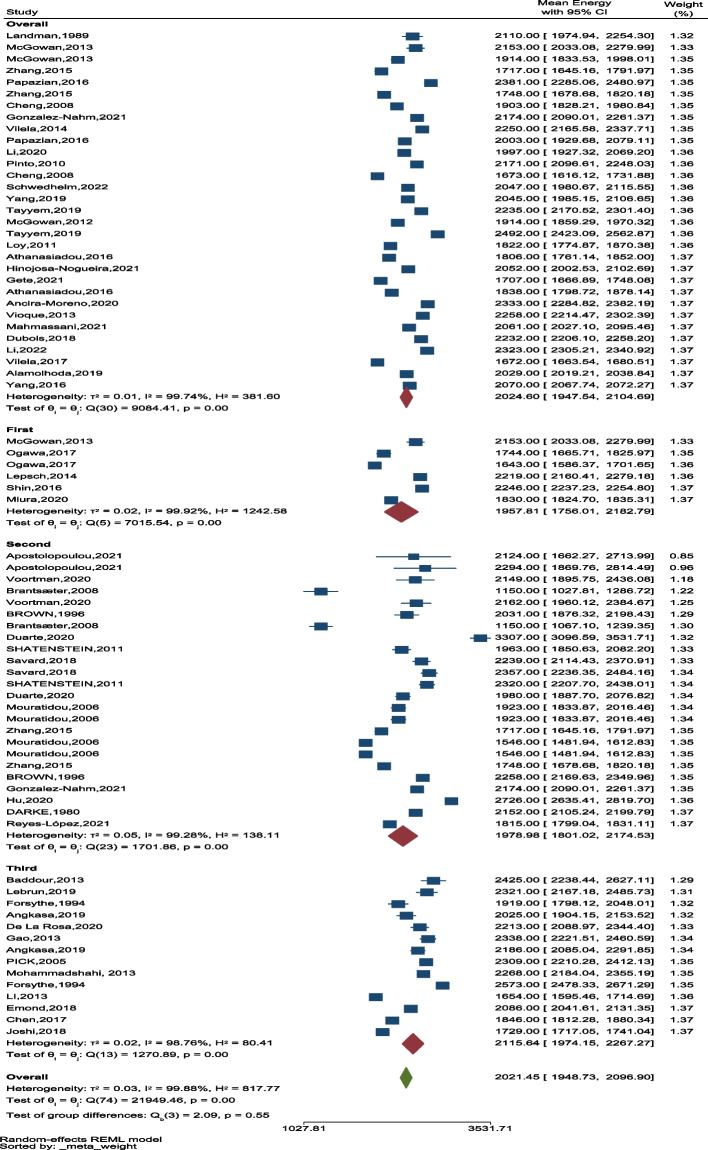
The forest plot of average energy based on 3 trimester

In the cohort studies, the lowest and the highest mean intake were reported in the case–control studies (Fig. 8). In addition, the overall average energy intake was the highest in the third trimester of pregnancy (Fig. 9).

## Discussion

This systematic review and meta-analysis summarizes the extant evidence related to the food-derived energy and macronutrient intakes of pregnant women in different countries.

This investigation revealed that total average energy intake of pregnant women was 2036.10 kcal/day (recommended energy intake during pregnancy is set at 200 to 300 kcal/day (FAO/WHO/UNU, 1985; NRC, 1989) above non-pregnant levels about 2200–2500 kcal/day) [73]. In this meta-analysis study, this average is lower than the recommended normal average. In addition, we found that, on average, an intake of 109 kcal/day and 49 kcal/day less than women from developed countries, middle and low -income countries, respectively [74–76]. Moreover, the range of energy intake in WHO regions was between 1867 -2228 kcal/day and the highest mean was in the American and Eastern Mediterranean regions. Our findings were consistent with two other reports [76, 77], but the range of energy in these studies was 7710 to 9260 kJ/day.

Contrary to the results of this study, in the meta-analysis study that was conducted in Indonesia and Malaysia, the average energy intake based on the type of study was the highest in cross-sectional studies (1895 kcal/day) and the lowest in case–control studies (1220 kcal/day). This may be due to the difference in the region, the number of studies of each type of study, and trimester of pregnancy [78].

According to our results, average energy intake was higher when using the FFQ questionnaire and the lowest in 24-h recall. This is similar to a previous study [79], however, in contrast with the Shatenstein study [23]. This difference might be due different regions, sample sizes, and study designs. Heterogeneity among countries is expected due to different levels of income, food access and food security. Diet can be influenced by several factors at the macro and micro level. The reported differences between countries could be the result of macro level factors (macro environment) referring to structures such as food systems (access to land and food production), nutrition policies and reforms, mass media and culture. The macro environment influences the micro level factors which in turn influence the diet of the population.

In this study, similar to a prior study, the third trimester of pregnancy had the highest daily energy intake compared to other trimesters [78]. Since energy intake is the main cause of weight gain during pregnancy, the mother's diet should be a sufficient source of energy to meet the usual needs of the mother, as well as the needs of the growing fetus, which includes the synthesis of new tissues (placenta, embryo and amniotic fluid) and the growth of existing tissue (fat tissue of the mother, breast and uterus) [80]. However, energy demands vary widely during pregnancy, so energy intake should be adjusted based on pre-pregnancy body mass index (BMI), metabolic rate, and physical activity level. In the last month of pregnancy, the fetus grows rapidly, which is associated with an increase in the weight of the fetus, and the mother's nutritional needs also increase. Another study showed that energy intake in pregnant women in the third trimester has a strong positive correlation with birth weight [81]. Contrary to these studies, Gennaro found that energy intake in the third trimester of pregnancy was not high [82], this could be because the consumption of nutrients among the participants in the third trimester of pregnancy was constant.

According to our results, the mean of protein intakes (78.21 gr/day) was higher than in the previous studies (52.4 g/d and 64.3 g/d) [75, 83]. The recommended amount of protein for pregnant women is 60 to 70 g per day [84]. This suggests that the protein intake of pregnant women may have improved over the past few decades. A simultaneous reduction in maternal urea synthesis, urinary urea excretion, and amino acid concentration, occurs in early pregnancy and remains low throughout pregnancy. In well-nourished pregnant women, these physiological changes conserve nitrogen and increase protein to ensure sufficient nutrients are provided to the fetus [85].

In addition, in a meta-analysis study conducted in the Eastern Mediterranean region [86], like our study, the intake of carbohydrates was high in most countries. In general, it is recommended that pregnant women should consume 175 g/day of carbohydrates [84]*.* The average carbohydrate intake in the present review (262.17 g/d) was similar to two previous reports (297,269 g/d) [74, 76] and more than normal range. Generally, these similarities and differences may reflect the impact of macro-level socioeconomic inequalities that affect access and choice of food options and shape the dietary patterns of populations [87]. The type of carbohydrates consumed (low or high glycemic sources) affects the fetus and mother during pregnancy. Eating high-glycemic carbohydrates leads to excessive maternal weight gain and overgrowth of the fetus-pair, while low-glycemic carbohydrate consumption increases the normal weight of the mother and produces infants with normal weight [88].

As observed in this and previous studies [86, 89], fat intake was found to be high in some countries. However, women in some developed countries showed slightly higher fat intake than women in Villanueva study and our study (86 vs 71 and 74 g/day) [74, 76]. It is recommended that approximately 40–90 g of fat needed each day [90]. A higher intake of saturated fatty acids, at the same time as a lower intake of other macronutrients (including refined sugar), is associated with an increase in infant fat mass. In addition, excessive consumption of saturated fats is associated with increased infant obesity, weight for age, and waist-to-hip ratio at six months of age, and causes obesity, insulin resistance, and cardiovascular diseases at older ages. Therefore women should be encouraged to limit saturated fatty acids especially [91].

Different reports among countries may be due to the differences in structures such as food systems (access to land and food production), different income levels, different dietary assessment methodologies culture, small sample sizes, mass media, and nutrition policies. Moreover, the environment can affect micro-level factors, which in turn affect the population's diet.

Maternal nutrition is a key factor in the intrauterine environment, necessary for fetal development. As an important modifiable factor, maternal diet can be easily intervened at low cost and low risk. Impaired maternal nutrition during critical periods of development may have long-term effects on fetal tissue development and is a risk factor associated with chronic diseases and metabolism in adulthood, including diabetes and cardiovascular disease.

### Limitations

There are limitations in this study that should be noted. The included studies used a combination of dietary assessment tools, which may introduce a measurement bias, including under or over reporting. We also faced challenges in using the collected information, where among the macronutrients, protein was reported more than fat and carbohydrate in different regions, which makes it difficult to estimate the distribution of macronutrient intake. In addition, multiple studies in individual countries may unreasonably influence the total nutrient intake of the region. In some studies, different specific dietary patterns were used that were not applied in this study. For the average of fat and carbohydrates, if two questionnaires were used, only one of the questionnaires was considered. One of the strengths of this study was the use of robust meta-analytical methods and systematic review guidelines, in addition to comprehensively considering studies carried out in different countries covering the 5 continents.

## Conclusions

In general, the average total energy in this study was less than the average total energy required during pregnancy. The importance of maintaining a healthy and varied diet during pregnancy should not be overlooked. Indeed, nutritional deficiencies during pregnancy, especially in deprived and high-risk populations, are still one of the biggest public health problems. Health policies should prioritize the establishment of sustainable food systems that enable healthy and sustainable food choices and promote healthy eating patterns to enable nutrient intake to meet the needs of the mother and of the baby.

### Supplementary Information

**Additional file 1.**

## Data Availability

All data and materials used in this research are available via the corresponding author on request.

## Abbreviations

WHO: World Health Organization
RCTs: Randomized controlled trial
BMI: Body mass index
SQ –FFQ: Semi-quantitative Food frequency questionnaire
24 h-R: 24- Dietary recall
FD: Food dairy
