# Supplementary material for: Maternal macronutrient and energy intake during pregnancy: a systematic review and meta-analysis
Source: BMC Public Health. 2024 Feb 15;24:478. doi: 10.1186/s12889-024-17862-x (PMC10870573; doi:10.1186/s12889-024-17862-x)
Supplement: Supplementary file 1 — Additional file 1. [file 12889_2024_17862_MOESM1_ESM.docx]

**Appendix1**

The search terms for PubMed were: (((dietary pattern[Title]) OR (diet quality[Title])) OR (food habits[Title])) OR (nutrition surveys[Title])) OR (food-frequency questionnaire[Title])) OR (diet record[Title])) OR (dietary recall[Title])) AND (pregnancy[Title])) OR ("maternal health"[Title])) and The search terms for Web of Science were: =((((((((TI=("dietary pattern")) OR TI=("diet quality")) OR TI=("food habits")) OR TI=("nutrition surveys")) OR TI=("food-frequency questionnaire")) OR TI=("diet record")) OR TI=("dietary recall")) AND TI=(pregnancy)) OR TI=("maternal health") and in SCOPUS database:( TITLE ( *"dietary pattern"* )  OR  TITLE ( *"diet quality"* )  OR  TITLE ( *"food habits"* )  OR  TITLE ( *"nutrition surveys"* )  OR  TITLE ( *"diet surveys"* )  OR  TITLE ( *"food-frequency questionnaire"* )  OR  TITLE ( *"diet record"* )  OR  TITLE ( *"dietary recall"* )  AND  TITLE-ABS-KEY ( *pregnancy* )  OR  TITLE-ABS-KEY ( *reproduction* )  OR  TITLE-ABS-KEY ( *maternal*  *health* )  OR  TITLE-ABS-KEY ( *"neonatal outcomes"* ) ) and in PROQUEST were: ti("dietary pattern") OR ti("diet quality") OR ti("food habits") OR ti("nutrition surveys") OR ti("diet surveys") OR ti("food-frequency questionnaire") OR ti("diet record") OR ti("dietary recall") AND ti(pregnancy) OR ti("maternal health") OR ti("neonatal outcomes")
